# A QUALITATIVE EVALUATION OF RISE FROM THE PERSPECTIVE OF ADULT PROTECTIVE SERVICES CASEWORKERS

**DOI:** 10.1093/geroni/igad104.1155

**Published:** 2023-12-21

**Authors:** Geoff Rogers, Andie MacNeil, M T Connolly, Erin Salvo, Patricia Kimball, Stuart Lewis, David Burnes

**Affiliations:** City University of New York, New York City, New York, United States; University of Toronto, Toronto, Ontario, Canada; University of Southern California, Los Angeles, California, United States; Department of Health and Human Services, Augusta, Maine, United States; Elder Abuse Institute of Maine, Brunswick, Maine, United States; Dartmouth College, Lebanon, New Hampshire, United States; University of Toronto, Toronto, Ontario, Canada

## Abstract

Our understanding of effective elder abuse and self-neglect (EASN) response interventions is limited. Adult Protective Services (APS), the primary agency responsible for responding to EASN, lacks a coherent, conceptually driven, prolonged intervention phase. Informed by ecological-systems, client-centered, and relational perspectives and adapting evidence-based modalities from other fields, the RISE intervention addresses this systems gap and compliments and augments APS services. Based on a three-year pilot project involving a partnership between RISE and Maine APS, the current study conducted a qualitative evaluation of RISE, from the perspective of APS caseworkers (n=14) who worked with RISE. The purpose of this evaluation was to understand RISE strengths, areas for improvement, and qualities of the RISE/APS partnership. Findings suggest APS workers perceive that RISE benefits clients, complements the scope and nature of APS, enhances APS caseworker well-being, and reduces repeat APS cases. Further APS/RISE collaboration and clarification on RISE role responsibilities and referral eligibilities represent areas of growth. This study provides preliminary evidence for RISE as a community-based EASN intervention in partnership with APS

